# Recent updates for antibody therapy for acute lymphoblastic leukemia

**DOI:** 10.1186/s40164-020-00189-9

**Published:** 2020-11-27

**Authors:** Le Li, Ying Wang

**Affiliations:** State Key Laboratory of Experimental Hematology, National Clinical Research Center for Blood Diseases, Institute of Hematology & Blood Diseases Hospital, Chinese Academy of Medical Sciences & Peking Union Medical College, Tianjin, 300020 China

**Keywords:** Acute lymphoblastic leukemia, Antibody–drug conjugates, T-cell redirecting antibodies, BiTE, Bispecific T cell engager, Blinatumomab, Bispecific antibody, Trispecific antibody

## Abstract

Acute lymphoblastic leukemia (ALL) is a hematologic malignancy arising from precursors of the lymphoid lineage. Conventional cytotoxic chemotherapies have resulted in high cure rates of up to 90% in pediatric ALL, but the outcomes for adult patients remain suboptimal with 5-year survival rates of only 30%-40%. Current immunotherapies exploit the performance of antibodies through several different mechanisms, including naked antibodies, antibodies linked to cytotoxic agents, and T-cell re-directing antibodies. Compared with chemotherapy, the application of an antibody–drug conjugates (ADC) called inotuzumab ozogamicin in relapsed or refractory (R/R) CD22^+^. ALL resulted in a complete remission (CR) rate of 81% and an overall median survival of 7.7 months with reduced toxicity. Similarly, blinatumomab, the first FDA-approved bispecific antibody (BsAb), produced a 44% complete response rate and an overall median survival of 7.7 months in a widely treated ALL population. In addition, approximately 80% of patients getting complete remission with evidence of minimal residual disease (MRD) achieved a complete MRD response with the use of blinatumomab. These results highlight the great promise of antibody-based therapy for ALL. How to reasonably determine the place of antibody drugs in the treatment of ALL remains a major problem to be solved for ongoing and future researches. Meanwhile the combination of antibody-based therapy with traditional standard of care (SOC) chemotherapy, chimeric antigen receptor (CAR) T-cell therapy and HSCT is also a challenge. Here, we will review some important milestones of antibody-based therapies, including combinational strategies, and antibodies under clinical development for ALL.

## Background

The application of classical multi-agent chemotherapy in patients with ALL results in CR in more than 80% of patients. About 50% of newly diagnosed patients can achieve long-term disease control with further intensification or maintenance therapy. However 10% have initial refractory disease [1, 2]. What’s more, many patients with ALL will subsequently relapse after remission from initial chemotherapy. Due to practical constraints, prognosis of R/R ALL remains grim. Treatment options are limited previously [3, 4]. Only 20–30% of these patients achieve a second complete remission with standard salvage chemotherapy [5].

Over 100 years ago, *Paul Ehrlich*, a German physician and scientist proposed the conception of antibodies as a “magic bullet” for selective targeting of malignant cells. Nevertheless, it took about a century to achieve the full potential of antibody therapy. Up to now, antibody-based therapies targeting leukemic cell surface antigens are major breakthroughs in the treatment of patients with ALL, changing the traditional treatment paradigms [6].

Based on the excellent outcomes in patients with R/R B-cell ALL, this magic bullet has actually been incorporated into the frontline. Antibodies against some tumor-associated antigens (TAAs) have performed well in clinical trials and have successfully come to fruition, such as inotuzumab ozogamicin [7, 8] and Blinatumomab [9, 10]; more and more potential sites is also in the process of demonstration and research. Antibody-based therapies are attracting considerable critical attention. In this review, we aim to present an overview of the efficacy and safety of the approved antibody-based constructs used for treatment of R/R ALL, and the ongoing researches of different formats, then give a brief introduction of combinational strategies.

**Fig. 1:**
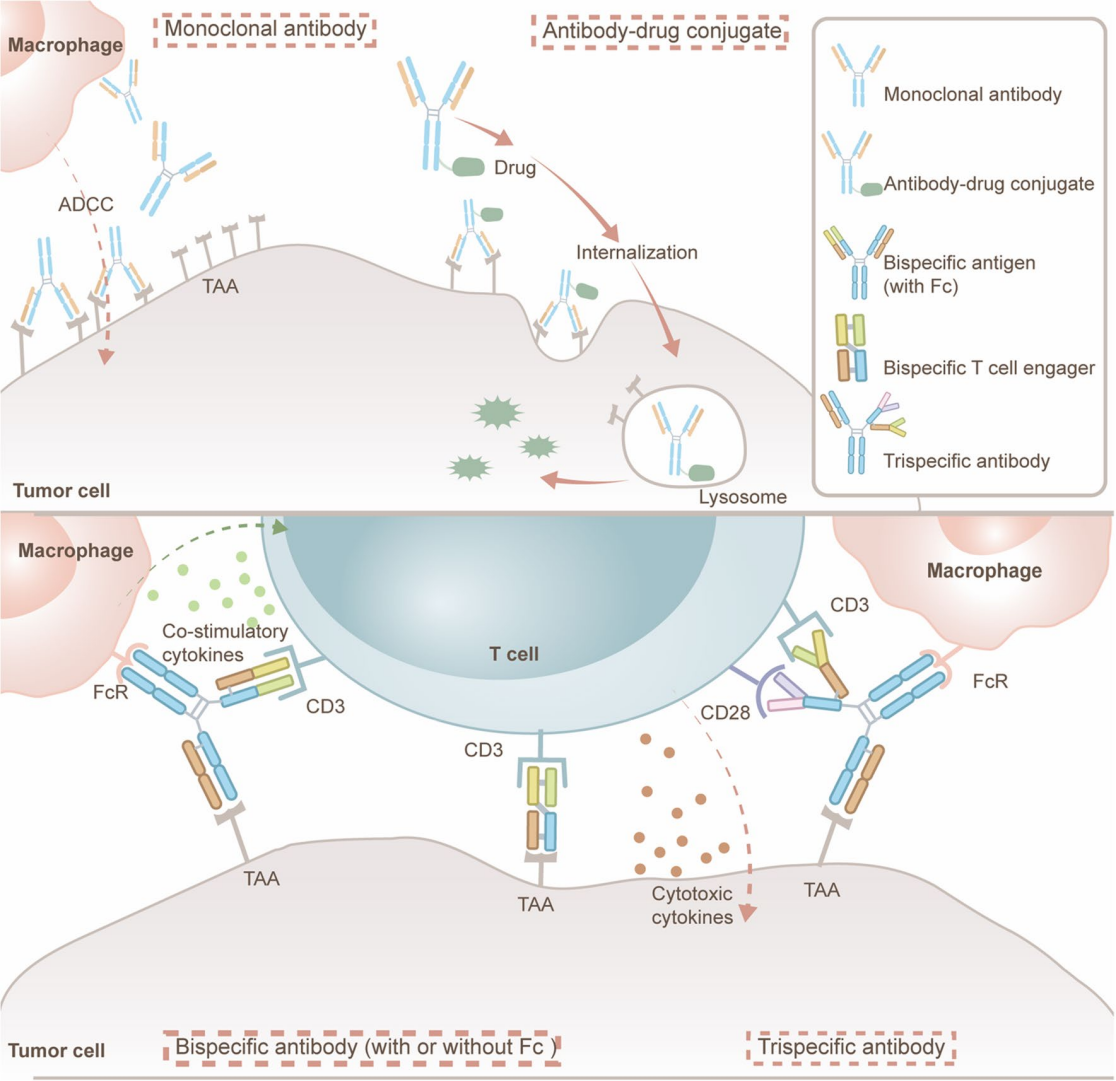
Schematic mechanisms of some popular antibodies. Classical monoclonal antibodies, antibody–drug conjugates, bi-specific antibodies (with or without Fc domain) and tri-specific antibodies. In addition, according to the Fc domain, BsAbs can be divided into two types: IgG-format molecules and non-IgG-format molecules (e.g. BiTE)

Monoclonal antibodies can be classified into three main groups according to their construction: naked antibodies, ADCs, and T-cell re-directing antibodies. These agents bind to known surface cell antigens present on the ALL blasts and mediate cell death through a variety of mechanisms that are specific to their target antigens and construct. Naked antibodies bind directly to the surface cell antigen and mediate cell lysis through antibody-dependent cellular cytotoxicity (ADCC), complement-dependent cytotoxicity (CDC) and induction of apoptosis. A variety of ADCs has also been developed that link a monoclonal antibody to a potent cytotoxin or radioisotope, knows as payloads. These conjugated antibodies are internalized upon binding to the surface cell marker, leading to cell death through the release of the toxic payload. Bispecific antibodies (BsAb) have attracted significant attention in antitumor immunotherapy [11]. Based on the structure of the Fc domain, BsAb can be classified into two types: IgG-like format and Fc-free format. BsAbs engage two different target epitopes and consist of variable domains linked together to form a single-chain antibody, such as BiTEs, dualaffinity re-targeting antibodies (DART) and tandem diabodies (TandAb). These antibodies lack the Fc region, therefore they are smaller in size. Although their half-life is shorter than other types of antibody constructs, they usually have better tissue penetration and lower immunogenicity. Additionally, bispecific antibodies (BsAbs) with a functioning Fc region, which can attract effector cells expressing FcR like macrophages, are called “trifunctional” (e.g. Triomabs). If BsAb has two or more binding sites for two different specificities, it is referred to as bivalent, trivalent or even tetravalent. Figure 1 schematizes brief mechanisms of different antibodies that will introduce in detail.

## Naked antibodies

Monoclonal antibodies for many established cell surfaces which are highly expressed on ALL blasts had achieved precise results. The CD20 antigen can be found in about 30–50% of B-cell ALL, while CD19 and CD22 are present on the cell surface in over 90% of B-cell ALL [12, 13]. Rituximab was first extensively studied in lymphoma and ALL [13, 14]. Several studies have evaluated its safety and efficacy with conventional chemotherapy [12, 15–17]. The addition of an anti-CD20 antibody, such as rituximab, to intensive chemotherapy in adults (aged below 60 years) with CD20^+^ precursor B-ALL is considered standard of care. In phase III multi-center randomized GRAALL-2005/R [17] trial, patients  < 60 years old with Ph-negative CD20-positive B- ALL were randomized to receive 16–18 doses of rituximab in addition to standard chemotherapy. The 2-year EFS was 65% in the rituximab group versus 52% in the control group (*p* = 0.04). The 2-year OS was 71% in the rituximab group versus 64% in the control group, although not statistically significant (*p* = 0.10). Sensitivity analyses with monitoring during HSCT showed a statistically significant improvement in OS in the rituximab group (hazard ratio, 0.55; *p* = 0.02). This study demonstrated that addition of rituximab to conventional chemotherapy improved the patient outcome with B-cell ALL in terms of EFS for a certainty. Ofatumumab is a second-generation anti-CD20 monoclonal antibody, that leads to more effective ADCC and CDC than rituximab, which is associated with its binding to a proximal small loop on the CD20 antigen [18, 19]. In an ongoing phase II study, ofatumumab has been similarly studied in combination with hyper-CVAD in newly diagnosed CD20 positive B-ALL patients (CD20 expression > 1%) [20]. However, whether ofatumumab improves long-term outcomes compared with rituximab in wider settings needs long-term follow up.

Given the fact that nearly 90% of B lymphoblasts in ALL express CD22, which rapidly internalized upon ligand binding [13], researchers are now focusing on it. Epratuzumab is an anti-CD22 monoclonal antibody with a very limited therapeutic effect. In pediatric patients, its effect in combination with chemotherapy is not obvious [21, 22], and is currently undergoing an international phase 3 trial (NCT01802814).

The latest analyses show that CD38 is a promising therapeutic antigen for acute leukemia [23]. Daratumumab, a recombinant anti-human CD38 monoclonal antibody approved by the FDA for the treatment of myeloma, has also been suggested to be effective in the treatment of acute leukemia. It not only exploits classical ADCC mechanisms depending on CD38 expression on tumor cells, but also plays a multifaceted [24] immuno-modulatory role (recently reported in MM patients) [25]. Evidence of the role of daratumumab in T-ALL has been demonstrated in preclinical studies using mouse models [26, 27]. According to the latest literature, there is one case of successful remission induced by daratumumab in a patient with Ph^+^ refractory B-ALL [28], and another one case report of eradication of MRD in a patient with advanced relapse after allo-HSCT [29]. The application of daratumumab for the eradication of MRD in high-risk advanced relapse of T-cell or CD19/CD22-negative acute lymphoblastic leukemia has been demonstrated. Cerrano *et.al* recently described [30] the clinical and immuno-modulatory effects of daratumumab in a 44-year-old relapsing T-ALL patient after allo-HSCT(according to published MM schedule [31]). The patient remained CR with MRD-negativity for 16 months after the application of daratumumab. Therefore, the anti-CD38 antibody is considered to achieve a better outcome in low tumor burden cases, similarly to blinatumomab in B-ALL [32].

## Antibody–drug conjugates

### As a warhead used in the clinical-stage for ALL: inotuzumab ozogamicin (IO)

CD22 is a 135 kDa sialoglycoprotein that is generally considered as an important B-lineage surface antigen. There are further studies conducted to better understand the immunobiology and metabolism of CD22 to aid in the development of CD22-directed therapies for the treatment of B-lymphoid malignancies. In a flow-cytometric cell surface expression study of 104 ALL cases, there was a significant positive correlation between CD22 expression and ALL at 96% (considering the expression of  > 20% in blast cells as positive) [33]. CD22 undergoes constitutive endocytosis into B-cells and is not shed into the microenvironment after antibody ligation, and it is then degraded in lysosomes and not recycled back to the cell surface. Therefore, research indicates that CD22 is an attractive target in the development of novel targeted therapies.

IO binds to CD22 and is internalized to release calicheamicin, a cytotoxic payload that binds to double-stranded DNA. Upon antigen binding, the ALL cell endocytoses IO and the acidic environment of the lysosome dissolves the linker protein, thus releasing the calicheamicin toxin intracellularly. In vitro studies have shown that cells required CD22 expression for the uptake of IO, but continuous saturation of the receptor was not a necessity for apoptosis, suggesting that multiple low IO dosages may be effective [34].

### Single-agent: an INO-VATE study

IO was subsequently compared with standard salvage in the INO-VATE study, a phase III study of 326 patients with R/R B-ALL [35]. All patients aged  ≥ 18 years with R/R CD22-positive ALL were randomly allocated in a 1:1 ratio to receive either IO or combination cytotoxic chemotherapy. IO was given at a 1.8 mg/m^2^ per cycle in a fractionated weekly dosing (0.8 mg/m^2^ on day 1 and 0.5 mg/m^2^ on days 8 and 15 per cycle). The chemotherapy regimens were either the FLAG regimen, a high-dose cytarabine-based regimen, or cytarabine plus mitoxantrone.

The CR/CR with incomplete hematologic recovery (CRi) and MRD negativity rate was significantly higher in the IO arm with CR/ CRi rates of 81 versus 29% (*p* < 0.001) and an MRD negativity rate of 78 versus 28% (*p* < 0.001) by flow cytometry. Compared with the SOC group, more patients who received IO underwent HSCT (41 versus 11%; *p* < 0.001). The median PFS for IO and for SOC was 5 versus 1.8 months (*p* < 0.001), and the median OS was 7.7 versus 6.7 months (*p* = 0.04), respectively. Significantly higher remission rates were observed in all patients regardless of bone marrow blast percentage, CD22 expression, prior HSCT, or karyotype, except for Ph-positive ALL patients who did not preferentially benefit from the 2 therapies. Hepatotoxicity was the most common event in patients treated with IO. Veno-occlusive disease (VOD) was reported in 15 patients (11%) in the IO group. Most cases 10–15 patients occurred after HSCT and the median time of development was 16 days (3–39). In August 2017, based on the data of the INO-VATE study, the US FDA approved IO (BESPONSA) for the treatment of adults with R/R B-cell precursor ALL (BCP-ALL).

### ADCs under clinical development for ALL

Currently, new antibody-based therapies are in the early stage of development, most of which target three major antigens, namely CD19, CD20, and CD22, but also CD25, CD123, and CD38. The vast majority of antibodies can bind to different cytotoxins (Table 1).

**Table 1:** Novel ADCs in early clinical research in ALL

Antibody	Target	Payload	Phase of trial	Setting	Identifier
ADCT-602	CD22	PBD-dimer toxin	I/II	R/R CD22^+^	NCT03698552
ADCT-402 (loncastuximab tesirine)	CD19	PBD-dimer toxin	I	R/R	NCT02669264
SGN-CD19A (denintuzumab mafodotin)	CD19	monomethyl auristatin F (MMAF)	I	R/R Ph^−^	NCT01786096
ADCT-301 (camidanlumab tesirine)	CD25	PBD-dimer toxin	I	R/R CD25^+^ AML/ALL	NCT02588092
IMGN632	CD123	IGN	I	R/R CD123^+^	NCT03386513

*AML* acute myeloid leukemia, *ALL* acute lymphoblastic leukemia, *B-ALL* B-cell acute lymphoblastic leukemia, *Ph*^* − *^ Philadelphia chromosome negative, *Ph*^* +*^ Philadelphia chromosome positive, *R/R* relapsed/refractory, *T-ALL* T-cell acute lymphoblastic leukemia

### Targeting CD19

Loncastuximab tesirine (also ADCT-402) is an ADC comprising of a humanized anti-CD19 antibody, stochastically conjugated through a cathepsin-cleavable valine-alanine linker to SG3199, a pyrrolobenzodiazepine (PBD) dimer-containing toxin. The mechanism of SG3199 for DNA crosslinking contributes to persistence in cells [36], and SG3199 has had picomolar antitumor activity against human hematologic tumor cells in *in-vitro* studies [37]. In preclinical studies, loncastuximab tesirine has shown potent dose-dependent antitumor activity against CD19-expressing B-cell malignancies in both *in-vitro* and *in-vivo* preclinical models [38]. A phase I study (NCT02669264) to assess the safety, tolerability, PKs, immunogenicity, and preliminary clinical activity of loncastuximab tesirine in adults with R/R B-ALL [39], demonstrated acceptable safety and tolerability profile in patients with R/R B-ALL. However, a formal assessment of the potential effect of loncastuximab tesirine was not performed in the trial because of the early termination of the study (slow accrual of dose escalation). one patient experienced DLT and three of all 35 patients (8.57%) received CRs as a response to loncastuximab tesirine, and two of the three patients had received previous CD19-directed therapy (Blinatumomab was discontinued due to its toxicity). Further investigation in clinical trials would be more encouraging.

### Targeting CD25

Human CD25, the α-chain of the heterotrimeric interleukin-2 receptor, is a critical component in regulating the immune system [40–42], and its expression is limited to activated T cells, B cells, and regulatory T cells (T-reg). The expression of CD25 on the surface of AML and ALL blasts is associated with the failure of induction treatment, increased risk of relapse, and short overall survival [40]. The concept and safety of targeting CD25 in malignancies have been established, and there is a large number of therapeutic approaches at different stages of development, including immunotoxins, radioimmunoconjugates, and ADCs [43]. Camidanlumab tesirine (ADCT-301) is an ADC comprising a humanized anti-CD25 antibody stochastically conjugated to SG3199, a PBD dimer mentioned above. Results of the data from part1 of a phase I study, though limited, that conducted at 11 centers across the US in patients with CD25-positive R/R AML or ALL (NCT02588092) have been recently presented [43–45]. No additional adverse events (AEs) of interest for camidanlumab tesirine were observed in this study. Remarkable outcome emerged that AEs of polyradiculopathy/Guillain-Barré syndrome observed with camidanlumab tesirine in the R/R classical Hodgkin’s lymphoma (HL) population were not seen in this study [43].

### Targeting CD123

CD123, the alpha chain of the interleukin-3 (IL-3) receptor, is the major low-affinity subunit of the IL-3 receptor and promotes high-affinity binding to IL-3 when co-expressed with the beta subunit. IL-3 is mainly produced by T lymphocytes, and it regulates the production of hematopoietic cells by stimulating cell cycle progression, differentiation, and inhibiting apoptosis. Early studies have shown that IL-3 plays a key role in the development of leukemia by allowing leukemia cells to escape programmed cell death and grow autonomously [46]. The potential of CD123-targeted drugs in ALL remains largely unexplored. Data on the association between CD123 expression and B-ALL are limited. Nevertheless, rewarding attempts have already been made [47, 48].

## T cell-redirecting antibody

### The first and only approved BiTE: Blinatumomab

BiTE is a relatively mature kind of BsAb obtained by ligating anti-CD3 single-chain Fv (scFv) with its counterpart of various anti-tumor cell surface antigens through peptides, which can simultaneously bind T cells to tumor cells and induce continuous attack of the target without T-cell apoptosis or anergy. Blinatumomab is the first FDA and EMA approved BiTE for the treatment of R/R ALL. It is a small (55 kDa) single-chain peptide that links two antibody variable regions directed against CD3 and CD19. Cytolytic synapsis formed, T cells can be activated without costimulatory molecules. Blinatumomab results in the proliferation of CD8-positive T cells with a predominance of cytotoxic CD8^+^ T effector memory (TEM).

### Single-agent: TOWER study and BLAST study

Blinatumomab has shown encouraging results in phase I/II clinical trials in R/R B-cell ALL, especially in the setting of low tumor burden [49, 50]. The pivotal phase III multicenter, open-labeled international study, TOWER, demonstrated obvious advances in adults with R/R B-ALL with higher CR rates (34 vs. 16%; *p* < 0.001), greater MRD negativity (76 vs. 48%) and longer median OS (7.7 and 4 months; *p* = 0.001) in comparison to SOC chemotherapy [51]. This benefit was seen irrespective of age, condition of prior therapies, previous HSCT, or the percentage of bone marrow blasts, but was more marked in the first salvage (median OS 11.1 vs. 5.3 months). For blinatumomab in Ph^−^ ALL [49] or Ph^+^ ALL [52], the two crucial factors that influence the CR rate are the number of prior therapies (salvage chemotherapy) and tumor burden (measured by the percentage of bone marrow blast cells). The two adverse events of interest are neurotoxicity and cytokine release syndrome (CRS), which are reported in 10 and 5% of cases, respectively.

Another significant result came from a multicenter, phase II BLAST study [32], aiming to evaluate the safety, efficacy and tolerability of blinatumomab in adult with MRD-positive BCP-ALL. All 116 patients were in CR (65 first CR) after ≥3 intensive chemotherapy treatments and MRD was ≥10^–3^. The primary endpoint was MRD negativity after one cycle. A total of 91 of the 113 evaluable patients obtained a complete MRD response of 78% (82 patients were attained after the first cycle). The medians of RFS and OS for the group were 18.9 and 36.5 months, respectively. The tolerability of blinatumomab was similar to that reported in previous trials. Based on these results, the regulatory authorities in the US extended the authorization of blinatumomab to MRD-positive BCP (Table 2).

**Table 2:** Safety and efficacy results from clinical trials of blinatumomab

Type	R/R Ph^−^			R/R Ph^+^	MRD^+^	
Ref	[44]	[43]	[45]	[46]	[48]	[49]
Regimen	BLN	BLN	BLNvs SOC	BLN	BLN	BLN
Capacity	36	189	405	45	21	116
≥ second salvage	21%	39%	45%	82%		36%
Response	CR/CRh: 69%	CR/CRh: 43%	CR: 34% CR/CRh/ CRi: 44%	CR: 31% CR/CRh: 36%	MRD response: 80%	MRD response: 78%
OS (median)	9.8	6.1	7.7	7.1	–	36.5
Grade3 + neurotoxity	16%	11%	9%	7%	19%	13%
Grade3 + CRS	6%	2%	5%	0%	0%	2%

*MRD* minimal residual disease, *CRS* cytokine release syndrome, *Ref.* reference number, *R/R* relapsed/refractory, *Ph* Philadelphia chromosome, *BLN* blinatumomab, *SOC* standard of care, *CR* complete response, *CRh* complete response with partial hematologic recovery, *CRi* complete response with incomplete hematologic recovery, *OS* overall survival

To date, several mechanisms have been addressed to explain resistance or relapse after blinatumomab strategies. These factors comprise the presence of extramedullary disease [53], higher leukemia burden on treatment, PD-L1 expression (54) and loss of CD19 expression on leukemic cells [55]. Given the superior activity of blinatumomab in R/R B-ALL and the MRD^+^ settings, current investigations are ongoing to evaluate blinatumomab as a component of the initial treatment strategy (eg, NCT03914625). Similar studies using blinatumomab in other settings are also in the pipeline; for example, post-transplant maintenance to prevent relapse after HSCT (NCT02807883), which has a parallel in the use of post-transplant TKI (tyrosine kinase inhibitor) therapy in Ph^+^ B-ALL.

### T cell-redirecting antibodies under clinical development for ALL

IGM-2323 is a CD20/CD3 bispecific IgM antibody designed to treat patients with B cell Non-Hodgkin’s lymphoma (NHL) and other B cell malignancies. Its potential antineoplastic activity manifests itself. Instead of binding to one or two TAA molecules on the surface of the cancer cell, IGM-2323 has 10 binding units to CD20 and one binding unit to CD3. Therefore, it can bind to CD20-expressing cancer cells with higher avidity in comparison with the IgG type. This may include those clinical circumstances in which CD20 expression has been reduced due to prior treatment with other anti-CD20 antibodies, such as rituximab. Two mechanisms are employed aimed at killing cancer cells. One is T cell-directed cellular cytotoxicity (TDCC) and the other is CDC. Compared to IgG format BiTEs, IGM2323 appears to induce less cytokine release, and less CRS, associated with TDCC. Currently, there is only one study reporting the safety and pharmacokinetics of IGM-2323 in subjects with R/R NHL. Therefore, its potential to cure more hematological malignancies, like acute leukemia, could be anticipated.

### Other potential forms of BsAbs

Due to the small size of blinatumomab, it can reach the T cells and target membranes quickly, which also leads to its rapid clearance from the circulation [56, 57]. Therefore, BiTE should be administered continuously and at high concentrations (15–28 gperday) to recruit and activate large numbers of suboptimal T cells to achieve the half-maximal target cell lysis [58]. The antibody is administered as a 4-week continuous intravenous (IV) infusion to maintain effective therapeutic serum concentration [56], which directly increases the cost of treatment. The single polypeptide chain structure that enhances BiTE antibody-antigen recognition comes at the expense of increased aggregation and decreasing protein stability [59, 60]. In response to these issues, dual-affinity re-targeting proteins (DARTs) have been developed.

DART consists of two Fv fragments that form two unique antigen binding sites when they heterodimerize. Fv1 consists of VH from antibody A and VL from antibody B, whereas Fv2 is formed of VH from antibody B and VL from antibody A. Unlike BiTEs antibodies which are linked via a polypeptide linker, this combination allows DART to mimic natural interactions within IgG molecules. Compared to BiTE, DART molecules can also maintain potency when administered both in vitro and in vivo [61, 62]. Moore et al*.* [60] compared the in vitro ability of CD19xCD3 DART and BiTE molecules to kill B-cell lymphomas and found that DART molecules consistently outperformed BiTE molecules.

Compared to natural antibodies, the renal clearance of small-sized scFv lacking the Fc region is significantly higher. Tandem diabodies (TandAbs) have emerged to address the size problem. These tetravalent bispecific antibody provides two binding sites for each antigen to maintain the avidity of each natural bivalent antibody [63, 64]. Furthermore, the molecular weight of TandAbs (approximately 105 kDa) exceeds the threshold of first-pass renal clearance, hence its half-life is longer compared to other non-Fc antibody constructs [63, 65]. Clinical trials are underway correlating with two TandAb format drugs—AFM13 (CD30xCD16) for NK cell recruitment and AFM11 (CD19xCD3) for T cell recruitment.

### Three is promising for T-cell redirecting therapy

With the evolution of antibody technology, CD28 co-stimulation provides a novel choice for therapeutic interventions. Recently, Wu et al*.* [66] published a development about tri-specific antibody. The antibody has three targets: cancer cells, receptors that activate T cells and costimulatory signals that promote long-lasting T cell activity against cancer cells. It is based on bispecific antibody technology, and the innovation lies in the introduction of costimulatory domain that promotes enhanced T cell activation, that is, simultaneous targeting of CD3 and CD28 molecules on the surface of T cells and CD38 molecules on the surface of cancer cells. CD28 is a member of the immunoglobulin super family (IgSF), which is expressed on the surface of naive T cells under physiological conditions and binds to B7 molecules on the surface of antigen-presenting cells (APCs), providing an important second signal for T cell activation. This plays a key role in T cell proliferation and the production of cytokines such as IL-2, ensuring T cells activate correctly in spatial–temporal patterns. Lack of the second signal from costimulatory molecules will leads to a state of non-response, also termed as anergy.

To confirm the enhanced activity from costimulatory domain of trispecific antibodies, the group made versions of different combinations of all three binding domains that were mutated and tested them in "humanized" model mice with human T cells and human myeloma cells. Functional targeting of the CD28 domain enhanced T cell activation above that showed using antibodies lacking this domain. The results found that both proliferation of T cells and expression of the anti-apoptotic protein Bcl-xL were significantly enhanced, supporting the authors' hypothesis, that is, having costimulatory signals would prevent T cell apoptosis, even at the lowest antibody dose tested. The method of introducing the costimulatory domain has also been applied in chimeric antigen receptor T-cell (CAR-T) therapy at the same time. The main limitation of this study is the cytokine release syndrome (CRS) resulting from highly stimulated immune system.

## Novel combinational strategies

### Ph-negative ALL

Currently, several novel antibodies can provide effective salvage therapy for patients with R/R ALL, with higher response rates and OS rates compared with conventional chemotherapy. Early addition of these new agents to the frontline can be considered promising to further consolidate the therapeutic efficacy of relevant patients, while reducing the number of cytotoxic chemotherapies required to achieve a durable response. Such regimens can improve tolerability and reduce treatment-related morbidity and mortality. In R/R cases, correlative combination studies are ongoing to improve the results of the single-agent application of Blinatumomab and IO. Some trials have corroborated this, especially for patients in first salvage [67]. Although HSCT currently remains the treatment of choice in the second remission, combination regimens with new agents have shown a strong potential for alternation. Encouraging results have been published to indicate the efficacy and safety of combinational strategies for Ph-negative ALL (Table 3).

**Table 3:** Important combinational studies for Ph^−^ ALL

Type	Frontline Ph^−^ ALL			R/R Ph^−^ ALL	
Ref	[64]	[65]	[66]	[67]	[68]
Rigemen	Sequential hyper-CVAD + BLN	Mini-HCVD + IO ± BLN	SWOG 1318: BLN + POMP	SWOG 1312: CVP + IO	Mini-HCVD + IO ± BLN
Capicity	27	64	31	48	84
Conditions	–	–	–	44% Salvage 1 38% Prior BLN 19% Prior HSCT	13% Primary refractory 40% CR1 duration < 1 year 23% Prior HSCT
Median age [range]	38 [18–59]	68 [60–81]	73 [66–84]	43 [20–79]	35 [9–87]
Response (CR/CRi)	100%	98%	66%	61%	80%
MRD negativity by flow cytometry	96%	95%	92%	–	80%
Duration	RFS 76% at 1 year	76% at 3 years	DFS 56% at 1 year	–	52% at 2 years
OS rate	89% at 1 year	54% at 1 years	65% at 1 year	Median 10.9 months	39% at 2 years

*Ph* Philadelphia-chromosome, *ALL* acute lymphoblastic leukemia, *CR *complete remission, *CRi* complete remission with incomplete hematologic recovery, *MRD* measurable residual disease, *OS* overall survival, *RFS* relapse-free survival, *mini-HCVD* mini-hyper-fractionated cyclophosphamide, vincristine, dexamethasone, *IO* inotuzumab ozogamicin, *HSCT* hematopoietic stem cell transplant, hyper-CVAD, hyper-fractionated cyclophosphamide, vincristine, adriamycin, dexamethasone, *CVP* cyclophosphamide, vincristine, prednisone, *SWOG* South West Oncology Group, *POMP* prednisone, vincristine, methotrexate, mercaptopurine

### Ph-positive ALL: chemo-free?

Blinatumomab was demonstrated in the phase II ALCANTARA study to be safe and effective in patients with Ph-positive ALL [52]. In combination with TKIs, mainly ponatinib, grouped with blinatumomab has been demonstrated to be safe and effective in a small case consisting of 15 patients from MD Anderson Cancer Center. The CR rate and molecular response was 50 and 75% respectively [68]. Ponatinib is a potential TKI for patients with Ph-positive ALL [69]; however, its hepatotoxicity limits its potential for cooperation with IO. Accordingly, researchers combine IO with less hepatotoxic TKIs, bosutinib for instance. In a phase I/II trial, patients with R/R Ph-positive ALL or lymphoblastic phase chronic myelocytic leukemia(CML), combination of bosutinib with IO strategy showed exciting efficacy [70]. It must be mentioned that patients with T315I mutations are not eligible for the criteria of the study. Patients were administered bosutinib at a dose of 300–500 mg with IO at a dose of 0.5–0.8 mg/m^2^ on days 1, 8 and 15, given in 4-weekly cycles. 14 patients, with a median age of 62 years, were treated in the study. Overall, 79% of patients achieved CR/CRi, and 91% of responders achieved a complete cytogenetic response, 73% achieved MRD negativity. No BCR-ABL was detected in 55% (6/11) of responders. The median OS and EFS were 8.2 and 8.1 months, respectively.

The GIMEMA group has recently updated their results from D-ALBA [71], the first chemo-free induction-consolidation protocol including the sequential use of TKI/steroid in induction stage and blinatumomab in consolidation stage for adult Ph^+^ ALL patients of all ages. The general story is that, 5 relapses have been observed (2 hematologic, 2 isolated CNS and 1 nodal), the 12-month OS and DFS are 94.2% and 87.8%. A significantly inferior DFS (61.4%, *p* = 0.01) was observed in IKZF1plus cases. So deep molecular response improved throughout therapy, but patients carrying IKZF1 plus remains a clinical challenge.

A recent exciting result via chemo-free induction and consolidation first-line strategy with dasatinib and blinatumomab [72] brings out confidence. The combination shows high incidences of molecular response and survival, but few toxic effects of grade 3 or higher in Ph-positive ALL. Of all 63 patients (median age 54 years; range 24–82) enrolled, a complete remission was observed in 98%. At the end of dasatinib induction therapy (d85), 29% of the patients had a molecular response, and this percentage increased to 60% after two cycles of blinatumomab, and higher after additional blinatumomab cycles. At a median follow-up of 18 months, overall survival was 95% and DFS was 88% Patients who had an IKZF1 deletion plus additional genetic aberrations (CDKN2A or CDKN2B, PAX5, or both [i.e., IKZF1plus]) had a lower DFS relatively.

## Conclusion and future challenges

Novel antibody-based drugs such as blinatumomab and IO are some of the most exciting and promising agents approved for patients with ALL. However, the present study has several limitations that should be noted. Firstly, the short duration of response and survival outcomes, and the efficacy which is mainly dependent on the percentage and density of the antigen expression, as well as the persistence of antigen expression after repeated immunotherapy exposure. This suggests that antibody-based therapies alone cannot treat ALL. Therefore, we still need HSCT, traditional chemotherapy, TKIs, and even CAR-T therapy to achieve comprehensive treatment of ALL.

To know that patients are likely to quickly progress with each therapy, we need to consider which therapy to use first or how to sequence or combine the therapies. Therefore, clinical trials of low-intensity comparing IO with or without blinatumomab in first-line and R/R Ph-negative elderly patients are encouraging [73–77]. Retrospective studies have demonstrated the safety and efficacy of combining blinatumomab with TKIs, specifically ponatinib, in patients with Ph-positive ALL, and several prospective studies of these combinations are ongoing in both frontline and R/R settings. Highly effective combination therapies may reduce the need for HSCT in first remission for some patients, especially if they can achieve a higher MRD-negative rate compared to conventional cytotoxic chemotherapy regimens [78, 79].

Besides, with the latest developments and approval of CAR-T cell therapy for ALL in recent years, how to rationally use the two new drugs has also attracted significant attention. Up to now, it is still not clear how prior blinatumomab therapy affects the ongoing anti-CD19 CAR-T therapy. There are also concerns that blinatumomab causes the loss of CD19 antigen and disrupted CD19 membrane export. However, remission of such a sequential application is still possible [55, 80, 81]. Finally, concerning the lack of an optimal target, an important limitation of antibody-based therapies need to be acknowledged, which is that the needs for patients with T-cell ALL remain unmet. Several studies are on the march to carry through this important clinical question. Novel therapies are needed for the early T-cell precursor subtype, which shows poor outcomes with conventional chemotherapy [82].

With the development of antibody-based therapies such as monoclonal antibodies, ADCs, bispecific, tri-specific, and even multi-specific antibodies, treatment options are widely expanded. As discussed above, antibody-based therapies still face challenges in determining the best-optimized treatment combination. However, successful clinical studies reporting combinations with other immunotherapies such as CAR-T therapy, and T-ALL with the few available drugs, is a reason to be optimistic that antibody-based treatment approaches may eventually become a success story in ALL therapy.

## Data Availability

The material supporting the conclusion of this review has been included within the article.

## Abbreviations

ALL: Acute lymphoblastic leukemia
BCP-ALL: B-cell precursor acute lymphoblastic leukemia
T-ALL: T-cell acute lymphoblastic leukemia
AML: Acute myeloid leukemia
CML: Chronic myelocytic leukemia
ADC: Antibody–drug conjugates
R/R: Relapsed or refractory
CR: Complete remission
CRi: Complete remission incomplete hematologic recovery
CRh: Complete response with partial hematologic recovery
OS: Overall survival
CRS: Cytokine release syndrome
BsAb: Bispecific antibody
MRD: Minimum residual disease
SOC: Standard of care
CAR-T: Chimeric antigen receptor T-cell
HSCT: Hemopoietic stem cell transplantation
CDC: Complement-dependent cytotoxity
ADCC: Antibody-dependent cellular cytotoxity
TDCC: T cell directed cellular cytotoxity
BiTE: Bispecific T-cell engager
DART: Dual-affinity re-targeting antibody
TandAbs: Tendem diabody
VOD: Veno-occlusive disease
IO: Inotuzumab ozogamicin
PFS: Progression-free survival
RFS: Relapse-free survival
DFS: Disease-free survival
Ph^−^: Philadelphia chromosome negative
Ph^+^: Philadelphia chromosome positive
PBD: Pyrrolobenzodiazepine
PK: Pharmacokinetic
HL: Hodgkin’s lymphoma
IL-3: Interleukin-3
scFv: Single-chain Fv
TKI: Tyrosine kinase inhibitors
APC: Antigen presenting cells
